# Comparative efficacy of tulathromycin and tildipirosin for the treatment of experimental *Mycoplasma bovis* infection in calves

**DOI:** 10.1002/vms3.31

**Published:** 2016-05-14

**Authors:** David J. Bartram, Hilde Moyaert, Bindu H. Vanimisetti, Clifford P. Ramage, David Reddick, Michael R. Stegemann

**Affiliations:** ^1^ Zoetis Mercuriusstraat 20 Zaventem 1930 Belgium; ^2^ Zoetis 333 Portage Street Kalamazoo 49007 Michigan, United States; ^3^ Moredun Scientific Pentlands Science Park Bush Loan Penicuik EH26 0PZ United Kingdom

**Keywords:** antimicrobial, bovine, macrolide, Mycoplasma, pneumonia

## Abstract

The objective of this negative controlled, blinded, randomised, parallel group study was to compare the efficacy of two injectable macrolide antimicrobials, tulathromycin and tildipirosin, administered by single subcutaneous injection at dose rates of 2.5 and 4.0 mg kg^−1^ bodyweight, respectively, in the treatment of an experimentally induced *Mycoplasma bovis* infection in calves. A total of 238 *M. bovis‐*negative calves were challenged on three consecutive days with *M. bovis* by endobronchial deposition. Post‐challenge, a total of 126 animals fulfilled the inclusion criteria and were randomly allocated to three treatment groups: tulathromycin, tildipirosin and saline. Clinical observations for signs of respiratory disease and injection site assessments were conducted daily for 14 days post‐treatment. The animals were then killed, the lungs were examined for evidence of lesions, and samples collected for bacterial isolation. Calves treated with tulathromycin had a lower percentage of lung with lesions (*P *=* *0.0079), lower mortality (*P *=* *0.0477), fewer days with depressed demeanour (*P *=* *0.0486) and higher body weight (*P *=* *0.0112) than calves administered tildipirosin.

## Introduction

*Mycoplasma bovis* is one of the main primary and secondary bacterial pathogens associated with the bovine respiratory disease (BRD) complex along with *Mannheimia haemolytica, Pasteurella multocida* and *Histophilus somni*, and constitutes a major source of both welfare and financial concern for the cattle industry worldwide Manusell & Donovan 2009). It is an important cause of respiratory disease and arthritis in feedlot cattle and young dairy and veal calves worldwide as well as being a causative agent of mastitis in dairy cattle (Maunsell *et al*. 2011). In Europe, *M. bovis* is considered to be involved in BRD outbreaks in one to two‐thirds of herds (Nicholas & Ayling 2003; Vangeel *et al*. 2011), although in the past, the difficulties with culturing the organism in the laboratory may have resulted in an underestimate of the actual number of cases confirmed.

Like all Mollicutes, *M. bovis* is inherently refractory to certain groups of antimicrobials, such as beta‐lactams, because it does not possess a cell wall, which limits the range of effective products available for its control. Commercially available *M. bovis* bacterin vaccines have poor efficacy for the prevention of *M. bovis*‐associated respiratory disease in calves (Maunsell *et al*. 2009; Soehnlen *et al*. 2011) although work to develop more effective vaccines is ongoing (Zhang *et al*. 2014). As a result, treatment and prevention of the disease in the field is limited at present to management strategies and antimicrobials. Evidence is accumulating that the susceptibility of *M. bovis* to antimicrobials is reducing (Nicholas *et al*. 2000; Gautier‐Bouchardon *et al*. 2014), potentially further limiting the range of effective products available.

Tulathromycin (Draxxin^®^, Zoetis) is a 15‐membered semi‐synthetic macrolide antimicrobial. Due to the unique chemical structure of the molecule, which has three nitrogen/amine functional groups, tulathromycin is the first member of a novel subclass of macrolides known as triamilides (Evans 2005). Tulathromycin is authorised by the European Medicines Agency (EMA) for the treatment and prevention of BRD associated with *Mannheimia haemolytica, Pasteurella multocida, Mycoplasma bovis* and *Histophilus somni*. The efficacy of tulathromycin in the treatment and prevention of *M. bovis* infections in cattle has been established in several studies (Godinho *et al*. 2005; Moyaert *et al*. 2012). Tildipirosin (Zuprevo^®^, MSD Animal Health) is a semi‐synthetic derivative of the naturally occurring 16‐membered macrolide tylosin. Tildipirosin is authorised by the EMA for the treatment and prevention of BRD associated with *Mannheimia haemolytica, Pasteurella multocida* and *Histophilus somni*. The *in vivo* efficacy of tildipirosin for the treatment or prevention of *M. bovis* infections has not yet been reported.

Macrolides in general are bacteriostatic and inhibit essential protein biosynthesis by virtue of their selective binding to bacterial ribosomal RNA. They act by stimulating the dissociation of peptidyl‐tRNA from the ribosome during the translocation process (Menninger & Otto 1982). Tildipirosin and tulathromycin are rapidly and extensively distributed to the respiratory tract followed by slow elimination (Menge *et al*. 2012; Villarino *et al*. 2014). Tulathromycin also accumulates in inflammatory cells, including neutrophils and macrophages (Villarino *et al*. 2014).

The objective of this negative controlled, blinded, randomised parallel group study was to evaluate the activity of tulathromycin in the treatment of an experimental *M. bovis* infection in calves and to compare against the efficacy of tildipirosin in the same model. It was hypothesised that the tulathromycin‐treated animals would have a significantly lower proportion of the lung affected with lesions (the primary efficacy variable) than negative control and tildipirosin‐treated animals.

## Materials and methods

### Animals

A total of 238 dairy calves (mainly Holstein x Friesian males), 10–28 days of age, were collected from commercial dairy facilities following confirmation that they were negative for *M. bovis* antibodies by enzyme‐linked immunosorbent assay (ELISA) on serum samples and for *M. bovis* DNA by polymerase chain reaction test (PCR) on deep nasopharyngeal swabs. The ELISA test was developed and conducted by a commercial laboratory (Biobest, Darwin House, Edinburgh Technopole, Penicuik, Midlothian, UK). PCR testing was performed as previously described (Ayling *et al*. 1997). The animals were penned individually and bedded on straw from arrival for the duration of the study in one of two adjacent open sheds. Animals within a shed shared the same airspace. On arrival, animals were administered a single subcutaneous injection of florfenicol (Nuflor^®^, MSD Animal Health) in the right side of the neck at a dose rate 40 mg kg^−1^ body weight to reduce the risk of concurrent BRD, and then, allowed to acclimatise to the accommodation for 2–4 weeks prior to onset of procedures. The range in acclimation period was because the study ran over two phases as all study animals could not be housed simultaneously.

### Bacterial isolate

The challenge bacterium, designated *M. bovis* isolate 956, was originally isolated in Italy in 2000 from a calf which was diagnosed with BRD. This isolate has previously been used as part of regulatory standard studies to assess the efficacy of tulathromycin (Godinho *et al*. 2005). The minimum inhibitory concentration (MIC) of both tulathromycin and tildipirosin against the challenge isolate was >64 *μ*g mL^−1^. The sensitivity of the isolate was determined using a modification of the Tanner & Wu (1992) method described by Godinho *et al*. (2005).

### Preparation of challenge inoculum

A 1 mL vial of the master seed stock of *M. bovis* isolate 956 was thawed at +37°C (±2°C) and inoculated into a 9 mL volume of pre‐warmed *M. bovis* heart infusion medium (HIM/MB). The broth culture was incubated for 24 (±1) h at +37°C (±2°C). After 24 (±1) h, the 10 mL volume was inoculated into a 90 mL volume of HIM/MB and incubated for 24(±1) h at +37°C (±2°C). After 24(±1) h, 30 mL of broth culture was used to inoculate 270 mL of HIM/MB and the 300 mL volume was incubated for 48 (±2) h at +37°C (±2°C). At this point, the titre of the challenge culture should be approximately 1–9 × 10^8^ CFU mL^−1^. A sample of the challenge broth pre‐ and post‐challenge was retained for quantification of bacterial concentration. The procedure was repeated for each challenge broth to be administered on consecutive days.

### Quantification of bacterial concentration

Briefly, 10‐fold dilutions were performed on a sample of each challenge material (both pre‐ and post‐administration samples), by adding 200 *μ*L culture to 1.8 mL of Mycoplasma broth (Oxoid) and titrating to 10^−7^. A 200 *μ*L aliquot of each dilution (10^−4^–10^−7^) was inoculated onto complete Mycoplasma agar (Oxoid). The plates were incubated in 5% CO_2_ in a humidity chamber at +37°C (±2°C) for 3–6 days, after which time the resultant colonies were counted at each dilution for each of the duplicate plates, and the CFU/mL of each challenge inoculum was calculated as follows. The concentration of the challenge was calculated as follows. For each dilution of the challenge, duplicate colony counts were recorded and the mean number of bacterial colonies was calculated. Where possible, the mean count from the lowest dilution which gives the most reliable value (ideally between 30 and 300 colonies) was used to calculate the challenge concentration (CFU/mL) according to the formula: CFU/mL = mean × dilution factor × 5.

### Study design

**Figure 1:**
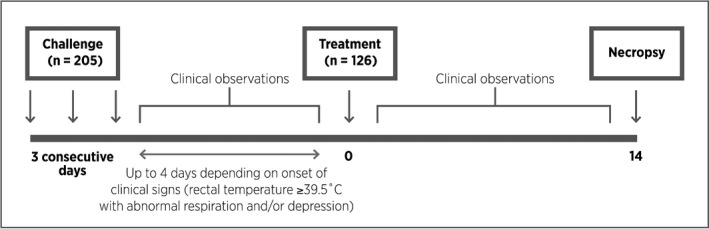
Experimental design.

The study design is illustrated in Fig. 1.

On Day 1, when animals were between 4 and 8 weeks of age, all animals were weighed and examined by a veterinarian to confirm suitability for inclusion on the study. A total of 205 animals fulfilled the inclusion criteria (negative for *M. bovis* and in good health with no evidence of respiratory disease) and were challenged on Days 0, 1 and 2 by endobronchial deposition, at the bifurcation of the main bronchus through a fibreoptic endoscope, with a mean concentration of 2.5 × 10^8^ CFU mL^−1^ (range 1.97 × 10^8^–2.92 × 10^8^ CFU mL^−1^ per animal) of *M. bovis* strain 956 in 12 mL of heart infusion media. Different challenge broths were administered on consecutive challenge days, but on each day, all animals received the same material to ensure all animals included in the study received an identical level of challenge. The animals were observed for 4 days following the final challenge and any which had clinical evidence of respiratory disease (increased rectal temperature ≥39.5°C with evidence of abnormal respiration (rate and/or character) and/or depressed demeanour) were enrolled into one of three groups, in an approximate ratio of 2:2:1. The study used a generalised randomised block design with the individual animal as the experimental unit. To accommodate sample size requirements, animals were enrolled in two batches 1 month apart. Within each batch, animals were housed in two sheds. Within a shed, animals were blocked based on the order of enrolment such that there were five animals planned in a block. Within each block, animals were randomly allocated to treatment groups – one for the saline and two each for the treated groups. Animals were assigned to treatment as they were encountered based on development of clinical signs according to the randomisation plan and as such, at enrolment, there were some incomplete blocks with fewer than the planned five animals.

A total of 126 animals were enrolled on the study and treated with either tulathromycin (*N* = 53), tildipirosin (*N* = 48) or 0.9% sodium chloride for injection (negative control) (*N* = 25). Tulathromycin (2.5 mg kg^−1^ body weight), tildipirosin (4 mg kg^−1^ body weight) and saline (1 mL/40 kg) were administered subcutaneously on the left side of the neck according to the manufacturer's instructions. Dose rates were based on body weights recorded on Day 1.

Post‐treatment, animals were observed clinically once daily for a total of 14 days. Clinical observations included the measurement of rectal temperature (°C) and assessments of demeanour and respiration (scored as absent, mild, moderate or severe as described by Godinho *et al*. 2005). A classification of ‘severe’ for either respiration or demeanour at any time after challenge resulted in withdrawal and premature killing of the animal on welfare grounds. Assessments of any injection site reactions (including measurement of vertical and horizontal diameters, mm) for all animals were recorded on a daily basis.

Fourteen days post‐treatment (or earlier if study endpoints were reached), animals were killed, and body weight was recorded. The lungs were removed from each animal and assessed for the presence of lesions both visually and by palpation with each of the lung lobes scored based on the percentage of affected lobe following the methodology described by Jericho & Langford (1982). Lung lavage samples were collected as previously described (Godinho *et al*. 2005) for bacteriological enumeration.

All unblinded procedures, such as allocation to treatment group and administration of test materials, were conducted by personnel who were not involved in any of the subsequent observations. All observations (clinical observations, necropsy and lung scoring, bacteriology, bodyweights etc.) were conducted by personnel who were blinded to the allocation. The blinding code was not broken for blinded personnel until all study‐related observations had been completed.

### Statistical analysis

Statistical analyses were performed using SAS Release 9.2 (SAS Institute, Cary, NC). All hypothesis tests were conducted at the two‐sided 0.05 level of significance. The percent gross involvement of lesions for each lung lobe was summarised and then weighted using the following percentages (based on ratios of individual lobes to total lung mass): left apical, 5%; left cardiac, 6%; left diaphragmatic, 32%; right apical, 6%; right accessory, 5%; right cardiac, 7%; right diaphragmatic, 35%; and intermediate, 4%. The weighted lung lobe values were then summed to yield the consolidated lung lesion score (percent lung lesions) for each animal (Jericho & Langford 1982). Arcsine square root transformed percentage of total lung with lesions, log10 transformed *M. bovis* concentration in lung lavage samples, arcsine square root transformed percentage of days with pyrexia or abnormal clinical signs and post‐treatment body weight, were analysed using a general linear mixed model with the fixed effects of treatment and random effects of batch, shed, batch by shed interaction, block within batch and shed, and residual. Pre‐treatment body weight was included as a covariate in the analysis of post‐treatment body weight. Differences in mortality were evaluated using Fisher's exact test since the generalised linear mixed model analysis failed to converge. Injection site reaction surface areas were approximated for each animal at each time point using the formula for calculation of the area of an ellipse, area = (π × vertical diameter × horizontal diameter)/4, and analysed using a general linear mixed model for repeated measures. All available data from calves that were killed or died before 14 days were included in analyses for all outcome variables. Transformed data were back‐transformed where necessary for presentation in the results. Least squares (LS) means, 95% confidence interval (CI) and treatment contrasts are presented.

### Ethics and standards of experimental conduct

All experimental procedures in this study were examined and approved by the Moredun Research Institute Experiments and Ethics Committee and conducted under the terms of licences issued by the United Kingdom Home Office in accordance with the Animals (Scientific Procedures) Act 1986, consistent with international standards of good clinical practice (VICH GL9) and in compliance with the standard operating procedures of Moredun Scientific.

## Results

### Primary efficacy variable

#### Percentage of total lung with lesions

The percentage of total lung with lesions by the end of the study was significantly lower in tulathromycin‐treated animals compared to tildipirosin‐treated animals (7%, 95% CI: 0–23% vs. 12%, 95% CI: 1–31%; *P *=* *0.0079) and both treated groups had significantly lower percentage of total lung with lesions than the negative control group (23%, 95% CI: 10–40%; *P *=* *0.0001 and 0.0049, respectively) (Table 1).

**Table 1:** Summary of clinical signs of respiratory disease and end of study bodyweights

Treatment	Depressed demeanour		Abnormal respiration		Other clinical signs of respiratory disease^a^		% days with pyrexia (rectal temperature ≥39.5°C)		Bodyweight at end of study	
	LS mean % days	95% CI	LS mean % days	95% CI	LS mean % days	95% CI	LS mean % days	95% CI	LS mean (kg)	95% CI
Tulathromycin	0.9	0–4.5	42.7	29.8–56.0	2.3	8.6–32.3	14.1	8.6–20.6	67.6	51.4–83.8
Tildipirosin	4.0	0.6–10.1	39.0	25.9–52.9	3.7	2.5–27.1	14.5	8.9–21.2	65.7	50.4–90.0
Saline	17.9	6.4–33.6	78.9	64.0–90.6	17.6	5.0–35.6	33.7	23.7–44.4	62.3	53.5–71.0

a For example, nasal discharge or coughing. CI, confidence interval; LS, Least squares.

### Secondary efficacy variables

#### Mortality

There were no BRD‐related deaths or welfare withdrawals from the study in the tulathromycin group compared to 8.3% (4/48) in the tildipirosin group and 12% (3/25) in the saline group. The differences between the tulathromycin group and both the tildipirosin and negative control were significant (*P *=* *0.0477 and *P *=* *0.0302, respectively), however, there was no significant difference between tildipirosin and negative control groups (*P *=* *0.6847).

### Clinical observations

The percentage of days with depressed demeanour was significantly lower in tulathromycin‐treated animals compared to the tildipirosin‐treated animals (*P *=* *0.0486) and for both treatment groups this percentage was significantly lower than for the negative controls (*P *=* *0.0004 and *P *=* *0.0147, respectively) (Table 1). For both tulathromycin and tildipirosin, the percentage of days with abnormal respiration was significantly lower compared to the negative controls (*P *=* *0.0001), but there was no significant difference between the tulathromycin and tildipirosin groups (*P *=* *0.6052). For both tulathromycin and tildipirosin, the percentage of days with other clinical signs of respiratory disease was significantly lower compared to the negative controls (*P *=* *0.0005 and 0.0031, respectively), but there was no significant difference between the tulathromycin and tildipirosin groups (*P *=* *0.3283).

The percentage of days with pyrexia (rectal temperature ≥39.5°C) was significantly lower for both the tulathromycin (14.1%, 95% CI: 8.6–20.6%) and tildipirosin (14.5%, 95% CI: 8.9–21.2%) groups compared to the negative control group (33.7%, 95% CI: 23.7–44.4%) (*P *=* *0.0001), but there was no significant difference between tulathromycin and tildipirosin treatments (*P *=* *0.8733) (Table 1).

### *M. bovis* recovery from lung lavage fluid

The mean concentration of *M. bovis* in lung lavage fluid was significantly lower in the tulathromycin group than in the negative control group (0.0159 vs. 1.678 × 10^6^ CFU mL^−1^, *P *=* *0.0066). By contrast, the difference between the tildipirosin‐treated group (0.812 × 10^6^ CFU mL^−1^) and the negative control group was not significant (*P *=* *0.4054).

### Body weight

After statistical adjustment for pre‐treatment body weight, the body weight of the tulathromycin group by the end of the study was significantly greater than in the tildipirosin and negative control groups (*P *=* *0.0112 and *P *= <0.0001, respectively) (Table 1). There was also a significant difference between the tildipirosin and negative control groups (*P *=* *0.0045).

### Injection site reactions

Injection site reactions occurred in animals from the tulathromycin and tildipirosin groups from Day 1 post‐treatment onwards (84.9 [45/53] vs. 91.7% [44/48], *P *=* *0.3650), but no reactions were observed in the negative control group. The mean surface area of the reaction was significantly greater in the tulathromycin group than the negative control group for the duration of the study (2136 mm^2^, 95% CI: 1681–2591 mm^2^ on Day 1 to 302 mm^2^, 95% CI: 83–521 mm^2^ on Day 14, *P *<* *0.05) while in the tildipirosin group, it was significantly greater up to and including 6 days post‐treatment (3169 mm^2^, 95% CI: 2687–3651 mm^2^ on Day 1 to 405 mm^2^, 95% CI: 204–605 mm^2^ on Day 6, *P *<* *0.05).

## Discussion

The objective of this study was to evaluate the activity of tulathromycin for the treatment of an *M. bovis* experimental infection in calves (Godinho *et al*. 2005) and to compare against the efficacy of tildipirosin in the same model.

In this model, cattle treated with tulathromycin had a lower proportion of total lung with lesions, lower mortality, fewer days with depressed demeanour and higher body weight 14 days post‐treatment than cattle administered tildipirosin. Tildipirosin was significantly more effective than saline in reducing lung lesion development at 14 days post‐treatment, as well as reducing mortality, depressed demeanour, abnormal respiration, pyrexia and other clinical signs of respiratory disease, but the efficacy of tildipirosin was not significantly superior to tulathromycin for any of the variables examined. Each of the veterinary macrolides has a distinct chemical structure which attributes unique pharmacokinetic and pharmacodynamic properties (Evans, 2005; Villarino *et al*. 2014) and may account for the superior efficacy of tulathromycin against *M. bovis* infection in this study.

Our experimental challenge model was successful in inducing *M. bovis*‐associated disease, as demonstrated by the proportion of mortalities or welfare withdrawals in the saline‐treated calves. The isolate was selected due to its proven pathogenicity in this model which has been shown previously to produce respiratory disease in young cattle to a consistent and reproducible level, within welfare limits acceptable to the UK Home Office (Godinho *et al*. 2005; Moyaert *et al*. 2012). While the challenge model uses an artificial delivery method, the clinical disease observed closely mimics the clinical signs and disease progression that are observed during a natural outbreak in the field in calves of this age range. The age of calves and time of killing after infection is consistent with other *M. bovis* experimental respiratory challenge models (White *et al*. 2012). Calves with clinical signs of respiratory disease were treated within 4 days following the final challenge. In the field, clinical signs of respiratory disease may not be identified as readily as in a study environment in which the animals have frequent clinical examinations. However, the antimicrobials concerned are used not only for therapeutic treatment of animals showing overt clinical signs of BRD but also for metaphylactic treatment of groups of animals deemed to be at high risk of developing BRD or to be in the preclinical stages (Nickell & White 2010).

A limitation of the study is that calves were necropsied at a single time point (14 days) post‐treatment. Necropsies at multiple time points would have enabled an evaluation of whether pathology would have progressed or resolved further or whether calves in different treatment groups would have eventually reached similar outcomes. Moreover, calves in the study were challenged with a single archived isolate. Use of alternative recent field isolates in any future studies would help determine whether the results are reproducible. A further study limitation is that tests were not performed for other bacterial or viral pathogens which may have contributed to lung lesions in some calves. The shared airspace in this study allowed the potential for continued challenge and reinfection of calves in one treatment group from calves in the other treatment groups. This may explain why, although there was a significant reduction in pathogen load in lung lavage samples in tulathromycin‐treated calves compared to the negative controls at 14 days post‐treatment, *M. bovis* was not completely eliminated from the lungs. Risk of reinfection could be minimised in field outbreaks of disease through the metaphylactic treatment of all animals in a shared airspace.

Pulmonary pharmacokinetics of tildipirosin and tulathromycin were not evaluated in this study because these are already well established (Menge *et al*. 2012; Villarino *et al*. 2014).

Natural outbreaks of BRD commonly comprise infections with several different bacterial and viral pathogens, so it is useful to consider the study in relation to commercial farming operations. Both of these antimicrobials are used routinely in the field for the treatment and prevention of respiratory disease in cattle, however, only tulathromycin has a licensed claim against *M. bovis*. The superior efficacy of tulathromycin compared to tildipirosin in the treatment of *M*.* bovis* in this study may contribute to the reduced risk for retreatment reported in a recent mixed treatment comparison meta‐analysis of antimicrobial treatments for undifferentiated BRD (O'Connor *et al*. 2013). The results of this study support the use of tulathromycin to treat clinical *M. bovis* infections or undifferentiated BRD in which *M. bovis* infection is implicated.

This study provides further confirmation of the *in vivo* efficacy of tulathromycin against a high‐MIC *M. bovis* strain. This observation has previously been reported by Godinho *et al*. (2005) and calls for cautious interpretation of *in vitro* sensitivity data for *M. bovis* when assessing the suitability of tulathromycin for use in a clinical situation because standardised antimicrobial susceptibility test methodology and validated clinical resistance breakpoints are not yet established for this pathogen (Gautier‐Bouchardon *et al*. 2014).

Given the clinical and economic importance of *M. bovis*, and in light of responsible use of antimicrobials, it is of utmost importance to carefully select the most appropriate antimicrobial when animals require treatment. To date, tulathromycin is the only veterinary‐use macrolide which is specifically authorised in the EU for the treatment and prevention of BRD associated with *M. bovis*. This is further supported by the results of this study.

## Conclusions

In this model, cattle treated with tulathromycin had lower lung proportion of total lung with lesions, lower mortality, fewer days with depressed demeanour and higher body weight 14 days post‐treatment than cattle administered tildipirosin. Tildipirosin was significantly more effective than saline in reducing lung lesion development at 14 days post‐treatment, as well as reducing mortality, depressed demeanour, abnormal respiration, pyrexia and other clinical signs of respiratory disease, but the efficacy of tildipirosin was not significantly superior to tulathromycin for any of the variables examined.

## Source of Funding

The study was funded by Zoetis, Zaventem, Belgium.

## Conflict of Interest

David Bartram, Hilde Moyaert, Bindu Vanimisetti and Michael Stegemann are employees of Zoetis, the marketing authorisation holder for Draxxin^®^ (tulathromycin). The study was funded by Zoetis, Zaventem, Belgium.

## Contributions

All authors conceived and designed the experiments. CR and DR conducted the experiments. BV conducted the statistical analysis. DB and HM prepared the draft manuscript. All authors reviewed and agreed the content of the final manuscript.
